# The Role of the Bacillus of Yellow Fever

**Published:** 1897-09

**Authors:** 


### Editorial.

### THE ROLE OF THE BACILLUS OF YELLOW FEVER.

The bacillus of Sanarelli seems to be comparable to the bacillus of Eberth. The constant accompaniment of a disease is by no means the cause of the disease. The disease known as yellow fever has many germs accompanying its progress, and possibly present in the incipiency of the fever. Many bacteriologists have searched for the germ or bacillus supposed to be capable of propagating the disease. We have a literature which has now assumed the proportions of a library on this subject.

Bacteriologists differ as well as doctors. The difference of opinion seems to be based upon difference of microscopic observation as well as difference of opportunities. The names of Freire, Findlay, Cremona, Guiteras, Sternberg, and Sanarelli are embodiments, so to speak, of these ideas.

Now, to state the comparison meditated in this article between the bacillus of yellow fever, and the bacillus of enteric fever.

The various claims recently made to have discovered and isolated the veritable bacillus *icteroides* are conflicting.

Koch * made the following statement: "A very characteristic example of the difficulty with which the determination of a (bacterial) species is surrounded is exhibited by the bacillus of typhoid fever. If this be found in the glands of the mesentery, in the spleen, or in the liver of a typhoid victim, there can be no question but that we are dealing with the real typhoid bacilli, since no other bacteria have hitherto been found that could be confounded with these. But when we are dealing with the determination of typhoid bacilli in the contents of the intestine, in soil, in water, or in dust, the circumstances are very different.

"In such places there are found numerous bacilli similiar to those of typhoid, which only a very skillful bacteriologist, and even then not with absolute certainty, can distinguish from the typhoid bacillus, since unmistakable and constant characteristics of the latter are as yet, wanting."

Many of the medical journals have given more prominence to the bacillus of Sanarelli and to his recent claims of discovery as made public in an address which has been published in

---

* Reference Hand-book of Medical Sciences. Supplement, Vol. IX., page 916, article by George B. Shattuck.

the Medical Record of New York, than they have ever done to the other investigators equally deserving of attention. The whole subject was brought before the American Medical Association, at its recent meeting in Philadelphia, where Dr. J. McFadden Gaston, of Atlanta read a paper before the section on State medicine entitled the "Present Status of Yellow Fever Inoculation."

He claimed for Domingos Freire the discovery of the germ of yellow fever and its clinical demonstration by culture and inoculation.

The government of Brazil has recognized the inoculation by Domingos Freire while the Uruguayan government has recognized the labors of Sanarelli.

The one has the advantage that it has been tested for about seventeen years, while the other has the glamour of novelty. Dr. George M. Sternberg has been investigating the subject for many years, but has failed to do anything that would entitle him to rank with Domingos Freire. He is now claiming to have discovered the germ of yellow fever himself and that Sanarelli has only found now what he had previously described.

In the midst of a controversy of this nature, it is necessary that the plain practitioners should take the view that either no germ exists or that the best way to settle the matter is to take the statistics of inoculation, and test their veracity and let the matter of cause rest for a while.

The great work of Jenner is to a great extent one of practical every-day observation, and no amount of investigation can prove a theory to be true unless the premises are well taken. The truth is more important than the claims of any one man, and the test of inoculation has been sufficient to establish it on a firm basis.

